# American Academy of Dental Science

**Published:** 1897-05

**Authors:** 

**Affiliations:** American Academy of Dental Science


### Reports of Society Meetings.

### AMERICAN ACADEMY OF DENTAL SCIENCE.

The regular monthly meeting of the American Academy of Dental Science was held at Young's Hotel, Boston, December 2, 1896, at six o'clock. President Andrews in the chair.

Paper by Dr. J. G. W. Werner. Subject: "Not Failure, but Low Aim is Crime."—Lowell.

(For Dr. Werner's paper, see page 310.)

Following Dr. Werner's paper the society discussed the subject of "Crown- and- Bridge-Work."

*Dr. Cooke.*—There is one crown that I have used quite a little which gives the strength of the banded crown, and at the same time gives all the advantages, as regards appearance, of the porcelain crown, and that is made by using a combination of the band and the English tube tooth. It is made as follows:

Use a band as usual; cut it off at gum line; solder on a piece of 27 gauge clasp metal. Grind Ash tube crown to place. Mark place on cap where hole in crown comes; drill hole size of tube wire, pass wire through crown and into root as far as it will go. Cut wire a little longer than needed; remove cap and porcelain crown. With wire hold cap and porcelain in contact. With wire in position, press Melotte's moldine into root end of cap and around pin and over edge of band. Use small amount of moldine, press upon asbestos board, so it will stay upright while soldering. Remove porcelain crown and put Parr's flux and solder around pin and heat up at once and solder. The moldine will hold the parts firmly in place, and the work can be done in a few moments, no delay for heating. Put the crown and cap together and hold firmly in contact. Heat in flame and flow sulphur around post and between porcelain and band. Set on root as usual.

The question of removable or permanent bridges is one that we have not had a final answer to as yet,—in fact, I do not know that it has been fully settled what a bridge is. It seems to me there is quite a variety of opinions on that point. One man considers a bridge a fairly large plate attached to certain teeth and having a bearing on the gum as well as upon the teeth; while others claim that such an appliance cannot be called a bridge. They say that in a permanent bridge the pressure must come entirely on the teeth.

I have been running over in my mind a list of the bridges that I have put on for several years, and I do not know of one that has been a failure, and they have all been of the kind called permanent bridges. I have never put on a removable bridge, and therefore my criticism and opinion in regard to them may not be worth anything. My experience with permanent bridge-work has been very satisfactory; at the same time, my success may be due to the fact that I have not attempted too much. I do not believe in picking out four roots and attaching twelve or thirteen teeth to them,—I would not expect such a structure to stand. That would be a poorly-selected case and could not be used to determine the success or failure of permanent or removable bridge-work. Then the shape of the roots must be considered in this kind of work. For instance, pointed roots are not intended to stand the shock of mastication.

The following method is useful in fitting crowns on badly decayed roots: Secure anchorage in the root by means of a screw-post, as, for instance, a How post, then build amalgam around it and restore the shape of the roots with amalgam. When hard, polish and fit the band as usual.

*Dr. Barker.*—Mr. President, if it is in order, I would like to say something about one feature of Dr. Werner's paper.

*The President.*—It is perfectly in order.

*Dr. Barker.*—And it is about that portion of the paper wherein he emphasizes the importance of a cheerful disposition, as though a cheerful disposition were something which might be had for the asking, as black hair, or a blonde or a brunette complexion. I have said for a great many years, and I believe you will agree with me when I say still, that the most important single factor in the life of any human being is disposition or inherited temperament. A cheerful disposition, or a morose disposition, or any kind of a disposition, is something which comes to us and which we cannot make. It comes to us by inheritance, and in absolutely no other way. When I make that statement very likely the minds of some of you jump to the old discussion which we have heard so much about, and which is still a favorite subject for debate, the question of free will and predestination; in other words, how much can we modify disposition once inherited? I believe in a certain way, and up to a certain extent, in free will; that is to say, I believe, as I suppose all of us believe, that we have the capacity to originate an impulse. If so, it may to that extent, perhaps, be said, we are all little gods. I pick up that apple; I will drop it or I will hold it, as I choose. No one can successfully deny, even if they go into a long and bewildering metaphysical discussion, that I did, in the exercise of free will, originate the impulse to drop the apple. To this extent, in this way, and in no other way, can a man modify his disposition. If I have inherited a disposition to look upon the dark side of things, I must struggle against it just as long as I live. It is like gravitation, always operating in the same direction, and you are forced to contend against it all the time. What parent is there here, who cannot tell pretty nearly what Mary will do under certain circumstances? He can predict almost to a nicety what Charlie will do in given emergencies, and in the case of either child almost never make a failure. But I need not enlarge. I simply arose to suggest that I do not think Dr. Werner realizes the extent of the task he marks out for some people when he asks them to cultivate a cheerful disposition. A disposition is something which you get from your mother, or your father, or your grandmother, and although we may seem to cultivate it out of us, or to cultivate something into us, and we succeed in individual cases, through the principle of originated impulse, a case of atavism occurs, we revert to the original type, our true disposition shows itself, and we never really get rid of it. So when you speak of changing this, or changing that, you must begin at the right place to reform, and the reforming of a disposition, gentlemen, must begin with your grandmothers and your grandfathers; you must change dispositions in just precisely the same way that I have adopted in changing the plumage of my pigeons, by mating the proper colors; but, as a matter of fact, when John and Mary fancy each other, they do not take into consideration what kind of plumage the birds are going to have. That is what we must do, if the human race is ever to be perfected,—there must be more judgment exercised in mating.

*Dr. Werner.*—I do not disagree with Dr. Barker as regards the effect of heredity on disposition, but we can improve some of our inheritances. The human race, in all probability, did not begin with Adam; science teaches us the ascent of man. We have done very well so far, and we can do better yet. The training of a disposition is just as essential, and should be as much a branch of education as is the teaching of A, B, C, arithmetic, or any other study.

*President Andrews.*—I will now ask Dr. Wilson to give his views with regard to crown- and bridge-work.

*Dr. Wilson.*—About two years ago I read a paper before the Academy on this subject, and as nothing has transpired since that time to change my views, anything I may say this evening will be somewhat in the line of repetition.

There is no one subject which has called forth such a diversity of opinion on the expression of such extreme views. It seems to me we are inclined to take extreme views in matters pertaining to our profession, and, at times, to be somewhat inconsistent. For instance, one man says, "A skilfully constructed and properly adapted bridge is one of the most elegant and cleanly operations in dentistry," while another says, "As a practical fact, permanent bridges are simply the nastiest things ever made to do duty as substitutes for nature's work."

Now, we have all seen bridge-work in the mouths of patients which could exemplify the truth or falsity of either of those statements. For instance, we have patients who are dainty, fastidious, and scrupulously clean, both in their person and appearance. We also have patients of naturally cleanly habits, who have the will and inclination, but lack the faculty of keeping their mouths clean. We also have a third class who are indifferent and do not attempt to clean their mouths. I have patients of refinement and education, in the fashionable walks of society, whose outward attire is in the height of fashion and neatness, who never remove their artificial teeth.

Now, it stands to reason that bridge-work in the mouths of the first class of patients I have referred to—viz., those of dainty and cleanly habits—will present a very different appearance from bridge-work worn in the third class, whose mouths are habitually in a filthy condition. Therefore the matter of cleanliness has largely, if not wholly, to do with the patients. Dr. Bonwill very truly says, "A bridge cannot be kept any cleaner than a plate that is removable, and I never saw one of the latter that did not have to be polished out of the mouth."

In my opinion permanent bridge-work, except small pieces, cannot, as a rule, be kept in a cleanly and wholesome condition, and is one argument against its use. With all our advanced knowledge in the care of devitalized teeth it must be admitted that occasionally we come across "dead teeth" that are not wholly amenable to treatment, and remain in a semi-inflamed and sensitive condition. There are also many cases where dead teeth, which if left alone would remain in a good and healthy condition, would, if subjected to the weight and pressure of bridge-work, become sore, painful, and useless, and the only remedy would be the removal of the work, which might have been put in only the week before.

The foundation upon which the bridge rests, and is retained in the mouth, like the weak foundation of a house, is an element of uncertainity and more or less doubt, and is another reason or argument against the use of permanent bridge-work. Porcelain teeth are always liable to fracture. It is almost always possible to bore a couple of holes in the backing and cement a new tooth in the place of the broken one. This can be done with safety and certainty if the conditions are favorable.

There are, however, very many cases where, owing to narrow spaces and peculiar occlusion, with perhaps very thin backing, it is necessary to use small and weak teeth, liable to fracture at any time. The repair of such weak teeth in the mouth is a temporary expedient at best, and therefore necessitates the removal of the bridge from the mouth to properly repair it.

Nothing can be more trying to a busy man, with his book full of appointments, than to be obliged to repair a broken bridge for a patient who is going away immediately.

I have not as yet been in this predicament, but always realize the possibility of such happenings, and rather than run the risk of being caught in that way I prefer to send those who wish bridge-work somewhere else.

I have in mind the case of a lady who came to me a few days ago. I put in her mouth a bridge consisting of a dummy second bicuspid, attached to a first bicuspid, Richmond crown. The second bicuspid had been lost in very early life; the space had closed up very considerably, necessitating the use of a very small and weak piece of porcelain. While the lady was in Europe, fortunately for me, the porcelain was broken off. It was repaired, very nicely too, by some dentist abroad, who punched a couple of holes in the backing and cemented the tooth in place, and it has remained in that condition for about a year, but still it is liable to break down almost any time. If it had broken while she was here in Boston I would not have felt justified in repairing it in that way. There is always something happening to such cases. While I am perfectly sure there are a large number of people wearing bridges which meet all the requirements as regards cleanliness, looks, and durability, yet, for the reasons that I have given, I have not made bridge-work for a good many years. I do not think there is any one reason that I can advance which, more than any other, has led me to abandon this work, unless it is because of the element of uncertainty about it. While I have no prejudice against bridge-work when it is properly constructed,—I think it is a good thing,—yet personally, I limit myself to small pieces.

*President Andrews.*—I repaired a case in the way described by Dr. Wilson four or five months ago. One of the porcelain facings in a crown broke off, and I drilled a couple of holes through the gold backing and into the gold crown, and after carefully grinding a tooth with long pins to fit, I cemented it in with Harvard cement, and it is doing good service.

*Dr. Draper.*—I would say that my belief is a good deal as Dr. Wilson has stated his. I do not believe in large bridges, and I do think that in small cases where the patient will take great care of them, they are very successful. I remember that quite early in the practice of bridge-work I saw a case that came from "headquarters,"—where bridge-work is supposed to have originated,—and this case was an upper and a lower denture, supported on four teeth in each jaw. At the time I saw it the upper bridge was all right, but the lower one had given way at the foundations so much that what was supposed to be a fixed bridge had, in little less than a year, become a removable bridge. The patient could take it out roots and all and put it back again at will, and consequently I replaced it by a plate.

I was once a little unfortunate myself in that kind of a case. I made a bridge which extended from a wisdom-tooth to a cuspid; all the intervening teeth were gone. I put a gold crown on the wisdom-tooth, placed in four dummies,—two molars and two bicuspids,—and anchored the anterior end into the devitalized cuspid by a pin cemented into the canal. That was several years ago, and I think on an average I see that case about once in six months. The anterior end has loosened a little and needs to be reset. I think that too long a space is the trouble with a great many of the fixed bridges,—if the supporting teeth do not loosen from the strain they have to bear, either the filling or something else will give. In regard to repairing bridges, I have been very successful in using the Bryant method of securing plate teeth to the backing. After smoothing the backing where the tooth has been broken off, I select the facing to go on, putting a little rouge on the ends of the pins to mark where they are to go, and drill through the backing, making quite large holes and countersinking them on the inner surface. I then cut a thread on the pins of the tooth, select nuts to fit, and cement the tooth in place; screw the little nuts into the countersunk holes, cut off the pins, and polish to the surface of the backing.

What I had to say particularly this evening was with reference to bridge-work without crowns. I have been troubled, as I suppose all of you have in cases where, by the loss of some of the bicuspids or molars on either side of the lower jaw, the anterior teeth wear away the upper incisors on their palatal surfaces, and many times we find the pulps of the upper incisors being encroached on by wear. It was in such cases that I have adopted a method which has given me a great deal of satisfaction. I have not seen it practised by any one else, and will try to illustrate it as well as I can. The bicuspids and molars are built up sufficiently to open the bite by pieces of pure gold burnished on to the grinding surfaces, with two or three pins fitting into holes drilled into the teeth, and the occlusion made correct by pieces of plate and solder melted on. Dummy crowns are then fitted between the teeth which have been tipped and soldered to the tips, making a continuous bridge. The tips are then set with cement. The palatal surfaces of the incisors may then be restored. There is another method of bridging without crowns that I have used for the lower incisors, where there are perhaps one or two centrals or laterals missing, and the other teeth are loosened through Riggs's disease or by the presence of tartar. After cleaning the teeth thoroughly I have used the method (I do not remember who is the inventor of it, only that I read it in the *Dental Cosmos*) of fastening all of the incisors by burnishing pure gold against the lingual surfaces of these teeth, and then drilling above the pulp little holes about the size of an ordinary tooth-pin right through the tooth against the pure gold backing. Having the holes all parallel, drill through the teeth and the backing too; then insert little gold screws that go right through the teeth and fasten into the backing with solder gold; then strengthen the whole bridge by running a platinized gold wire along the back and flowing solder over it. If any tooth is missing a facing may be soldered to this backing. The bar and screws should then be placed in position with cement between them and the teeth. Next put the nuts on the outside and screw them up until the bar on the lingual surface is as tight as required. The nuts are removed after the cement has hardened, and the ends of the screws can be polished off so that they look like small fillings. The patient is only too glad to have the teeth saved, and does not object to this small disfigurement. I have here two or three specimens that show the inaccuracy that is sometimes used in fitting crowns that are put on, but do not touch the necks of the teeth either because of the teeth being not sufficiently trimmed, or from some other cause,—lack of skill, possibly. Here is one in particular that does not resemble a tooth any more than it does a percussion gun-cap. Here is a bridge that is something like the one I described. This was attached to a molar and a cuspid, but the cuspid loosened in the socket, and, of course, the rest of the bridge was of no value. The span was too great and something had to give way.

*Dr. Belyea.*—I have had a different experience from Dr. Cooke, but I have not seen a case of bridge-work that was properly constructed that has failed. Wherever there has been a failure of my work, it has been because I did not know enough to do it properly. I think bridge-work is all right, though I will admit there is a great deal of room for improvement in the construction of it. I have here some few ideas which I have stolen from various people. The first one was given to me by my friend on the right, Dr. Cutter. He told me a little while ago of his method of securing the measurement of a root and a pattern at the same time. He has a number of different-sized copper bands made extremely thin, and they match the various sizes of the S. S. White Dental Manufacturing Company's seamless collars. He fits the copper bands on the root to be crowned, and uses that for a pattern to cut his gold by. I took that idea and used it a little further. My first advance was to fit the band in the way he suggested, run it up as far as I could on the root, covering the end of the root with moldine, and getting from that a model that was almost a perfect representation of the root. Upon that I could fit a band or do anything I wished. I find it is a good deal more convenient to have a model to do the work on, so now in many cases I take these bands, fit them under the gum the required distance, take a plaster impression of the end of the root, remove the band, and then take a model of the root with fusible metal, and I have very nearly a perfect cast to work on.

Another person from whom I have taken the liberty of purloining some ideas is Dr. Hollingsworth. There is a method called the Hollingsworth method of making crowns. I think the best thing about it is the pamphlet. From that you get excellent ideas, and one of those ideas is to burn the band into a piece of wood. Another thing that I wanted to refer to is the making of cusps, the grinding surfaces of teeth. I have taken a little plan of my own for that. Take a couple of natural teeth and invest them in a section of brass tubing, a right and left, articulating surface protruding about one-eighth of an inch, take an impression of them in some modelling composition, and fill in with plaster. These cusps are susceptible to change,—you can do anything with them that you can with the Hollingsworth and more. After you have them in place on your model, you can articulate it, take the impression in moldine, and swage up your articulating surfaces.

Another way of using these teeth if you want to get a counter-die the same as the die-plate, only a good deal better, is to take a piece of brass bar rod, cut off the size you want, put it into the rubber ring, and pour in fusible metal while it is hot; after it has hardened you will get a counter-die which is absolutely perfect, and you can work on that eighteen- or twenty-carat gold without seriously marring your die.

Instead of making a plaster cup, another way is to take these dies as you have it shown there and swage one of shot. You can trim the lead so readily with a sharp knife that you can modify its shape almost as easily as you can plaster. The cause of most of the failures that I have had was the lack of stiffness of the gold that I used for bands and crowns; and I owe something to Dr. Wilson for recommending here one evening a few years ago the use of eighteen-carat gold for such purposes; but I think I have improved on that by using in some cases clasp-metal, which also has the advantage of being inconspicuous. I think Dr. Piper knows something about this bridge-work which Dr. Draper has drawn on the board.

*Dr. Piper.*—I suppose Dr. Belyea refers to the bridge which Dr. Draper drew on the paper showing the ends fastened into the teeth by means of pins. He got up this bridge for me and made valuable suggestions in regard to its construction, which I followed. It has done good service. I put it on about two years ago, and when I saw it a few days ago it was in perfect condition. I have also used a method similar to that which Dr. Draper referred to for fastening loose teeth. The case which I had was fastened by means of pure gold pins going through the teeth. It consisted of two centrals which were so loose that it was necessary to ligate them in order to hold them in place while being worked upon. Drilling through just below the cutting edge of each tooth from cuspid to cuspid and then through a narrow band of gold swaged to fit the lingual surface of each tooth, I then, into this band, soldered twenty-four-carat gold pins, setting the whole in place with cement and riveting down the pins. After four years of wear this appliance was found to be doing good work.

*Dr. Belyea.*—I had hoped that Dr. Piper would mention, relative to the case of which he first spoke, something that he noted after the use of it for a time. In addition to putting on the bridge he had to build up the other teeth in order to open the bite and to save wear on the other teeth. I believe he was supplying three teeth on each side.

*Dr. Piper.*—The bicuspids and sixth-year molars.

*Dr. Belyea.*—And he noticed after it had been in wear a little while, about a year, that the old occlusion had been re-established. From that and other things which I have noticed, I have learned that you cannot depend on one or two, or even four teeth sustaining the bite. Where there is constant pressure on a few teeth, those teeth will shorten in their sockets to bring about a general occlusion, and so when I am putting on a bridge which is a trifle long and have not been able to get the occlusion correct for any reason, I do not hesitate to set it and assure the patient that it will soon settle into place. You will sometimes see that in bridges where the dummies are supposed not to come in contact with the gum; after they have been in wear a little while you find that the gum hangs down over the dummies which were not made to touch.

*Dr. Piper.*—In regard to the case which Dr. Belyea just referred to, it is some four years ago that it first came to my notice. The patient was an elderly gentleman from New Hampshire, and at that time had worn the six anterior upper teeth nearly halfway down. After looking the case over I decided to open the bite by building down the front teeth with gold and putting in two bridges carrying the bicuspid on either side, attaching to the sixth-year molars by means of shallow caps and open-faced caps on cuspids; molar caps having four pins and cuspids, two running into the teeth about the gum line. Dr. Belyea suggested more than one pin, so I made them according to his ideas. For lack of time the front teeth were not built down, consequently he was obliged to do all his chewing on these two bridges, with the result of bringing the teeth into the same position, after four years of wear, as they were before the work was done.

*Dr. C. T. Barker.*—One thing I have not heard spoken of here to-night, and that is, placing on crowns without bands. In the majority of cases it is unnecessary to put on a band. I think I place on single teeth fewer and fewer bands every year. Patients do not like to have bands put on; in the first place, because the process is very uncomfortable, and, besides, the appearance of gold is objectionable, and I have found that a case is just as useful without the band. I never had one break.

Another thing is about setting the crown. I always used to set them with cement,—I think almost everybody does,—but lately I have set them with gutta-percha, and when for any reason it does become necessary to remove a crown it is very easy to do it, whereas if it is set with cement it cannot be done without destroying the tooth. It is a little more difficult to get accustomed to putting it on with gutta-percha, but in the end it is much more satisfactory.

*Dr. Smith.*—Will Dr. Barker please tell us how he would set with gutta-percha a bridge supplying, say, three teeth,—a molar and two bicuspids?

*Dr. C. T. Barker.*—When I spoke I referred to single crowns. Use the pink gutta-percha or base-plate, and have the tooth and gutta-percha both very warm. Of course, you have to be very careful to use the right amount of gutta-percha.

*Dr. Wilson.*—When you put it on, is the pin attached to the tooth, or do you place it in separately?

*Dr. C. T. Barker.*—With the pin attached. Soften the gutta-percha with cajuput oil and perhaps put the pin in cajuput oil. It will go into place if you have the right amount of gutta-percha, and being very warm and everything very dry, the gutta-percha will squeeze out and form a very thin covering over the end of the root.

*Dr. Belyea.*—I think I can enlighten Professor Smith on that matter. I set a great many of my bridges and nearly all of my single crowns with gutta-percha. I use Doherty's white base-plate gutta-percha; put that around the pin and around inside the band. This is forced into place while the root is yet moist so that it can be readily withdrawn and the surplus trimmed away. Then after preparing the root, dry it and clean it thoroughly, put in a little chloro-percha and set your crown in place. If you have a bridge you can set it equally well.

William H. Potter, D.M.D.,
*Editor American Academy of Dental Science.*

